# Mr. Hume, on Modern Pharmacopœias

**Published:** 1805-03-01

**Authors:** Jos. Hume

**Affiliations:** Logn Acre


### *To the Editors of the Medical and Phyſical Journal.*

GENTLEMEN,

THE justness of my former "Observations on modern Pharmacopœias," I am well assured, still remains valid. The subject is certainly of too much importance to society to be hastily deserted as incurable, while so many palpable errors, so much incongruence and ambiguity exist. I trust, therefore, I shall stand fully excused, if I still persevere in an humble endeavour to inculcate the necessity of that reform, which, if it do not reach perfection, may, undoubtedly, obtain uniformity and precision.

My first remarks, inserted in the Medical and Physical Journal for April, were, for reasons then given, principally confined to the last edition of the Edinburgh Pharmacopœia; and those which appeared in your Number for July, were, with but few exceptions, dictated by a superficial, though, as far as I have gone, I hope, a very accurate, reading of Doctor Swediaur's *Pharmacopœia Medici Practici Universalis.*

The contents of the letter I am now to lay before you, do not occur from any industrious, curious, or methodical examination of this or that Dispensatory, for such a task would occupy more time than I can well bestow; they result rather from contemplating the present condition of modern productions on this subject, in which I observe more discordance than the present highly advanced knowledge of the sciences ought to allow. But, above all, the dangerous disparity in our metropolitan dispensatories, those of the Colleges of London, Edinburgh and Dublin, demands our first consideration. Among these works I observe such a degree of dissonance, such faltering and hesitation, and so much more attention bestowed upon the the capricious nomenclature of the day than to the more essential parts of pharmacy, that nothing more is requisite to prompt me to confine these observations chiefly to vernacular errors.

Repeated examples have convinced me, that the major part of medical practitioners in the three kingdoms do not suspect that so much variance prevails. It were superfluous to urge, that, by virtue of authority from the King and Council, the jurisdiction of the College of London extends in some parts a few miles *beyond* the Tweed; and that the limits of the College of Edinburgh, also in its turn, reach still farther on *this side* of the same river.

Indeed, no two counties, or parishes, in the whole empire are more contiguous nor closely linked than England and Scotland; so nearly, indeed, that if the dispensatories be strictly obeyed, it may frequently happen, that the medicine prepared from the same prescription shall essentially differ when followed by one apothecary, who lives perhaps but a few miles, nay even a few yards, separate from another.

Is it not, therefore, truly absurd that one college should order more or less of opium, mercury, scammony, or other formidable article, or indeed of any acid, alkali or drug whatever, than is prescribed by so near a neighbour as the sister-college, in any composition to which *both* colleges give the *same* identical title? Is it consonant with the safety of medical practice for the *opium pill* of one of our dispensatories to exceed that of either of the others so much as to contain *double* the quantity of opium?

When any medicine has proved successful, either in the relief or cure of a disease, it is very natural, nay, very prudent, for the patient to be tenacious of such a prescription, to preserve it in case of a recurrence of his complaint, and to carry it with him wherever he may go. Those, therefore, who, either for pleasure or business, occasionally change their residence, particularly members of the united Parliament, should be very circumspect; they should be aware in the present imperfect state of the British dispensatories, that a mere change of place may produce a most serious change in the prescription; so that, what formerly had a happy effect, may now prove either ineffectual or dangerous.

Whenever either of the three colleges shall commence a reform, or, as it is commonly termed, a *new edition,* it will then be in its power liberally to come forward, and endeavour to converge towards that desirable *uniformity* of of *prescription,* if nothing more can be obtained;—then would be a favourable opportunity to change the proportion of this or that ingredient in some of the most objectionable compounds as they now stand; and, did no other reason exist, the change would be fully warranted were it merely the propriety of being in unison with a neighbour so near.

Such seasonable occasions have frequently presented themselves both to the College of London and to that of Edinburgh; and, I am sorry to observe, neither of them has availed itself of any such advantage. One of the most fair and opportune occurred lately, when the College of Edinburgh compiled the present edition of its Pharmacopœia.

A mere reduction or increase of a single compound, a slight change of an *opiate* or *mercurial pill,* of a *compound powder of scammony,* or of an *opiate electuary* or even of a *diluted sulphuric acid,* so as to assimilate with the standard of the London College, could not have disparaged the College of Edinburgh.

An example of such magnanimity, from the first school of medicine in the world, could not fail to be gratefully acknowledged and readily followed. Such conduct must have stimulated the other colleges to vie with each other to promote a coalition of sentiment as well as interest; and to fraternise, as it were, the whole medical kingdom.

If we compare some of the *formulæ* in the three Dispensatories, one would imagine they had been dictated by caprice; that each college was too tenacious of its own rights to make one step towards that mutual improvement, so essential to the success and accuracy of the practice of physic; and that, rather than forego that power, they would still continue in the line of deviation.

Even in one very simple article alone, sufficient grounds for this accusation may be found. The *diluted sulphuric acid,* a thing frequently prescribed, contains, according to one college, a *ninth* part of acid, and the other colleges have ordered one *eighth;* and this difference, although but slight, seems adopted without any reason.

The College of Edinburgh has, in some measure, I presume, invaded the nomenclature of its neighbour, by adopting the *same name* to denote a *caustic* article, which has, for these sixteen years past, been in use by the College of London to distinguish *mild* volatile alkali. In this age of novelty, when the propensity to coin new names is so predominant, some other more appropriate title title might have been devised in the place of aqua ammoniæ. Rather than encroach upon the London Pharmacopœia, one of these might have been preferred, viz. ammonia *soluta*—ammonia *aquosa*—*solutio* ammoniæ—or even the word *ammonia* alone. But why should the word *aqua* be joined at all? To define the article in question, is it not a *permanently elastic fluid* or *gas, dissolved in* water? Have we not many cases, perfectly analogous, in which we omit the word *aqua*? Is either the muriatic or nitric acid any thing more than a *gas dissolved* in water? So much, indeed, do I feel myself inclined to prefer brevity, that I should gladly consent to drop even the word *acidum* in the composition of names, both in chemistry and in pharmacy; for we might with the same propriety say, *alkali* potassæ, as *acidum* muriaticum.

There seems no apology for a species of versatility which modern writers adopt in their nomenclature. This may be clearly evinced by a few examples from the Edinburgh Pharmacopœia.

An aqueous solution is, in some instances, termed *solutio*; and in others *aqua*.

Thus *aqua* carbonatis potassæ, *solutio* muriatis calcis; *aqua* carbonatis ammoniæ, *aqua* acetitis ammoniæ, *solutio* sulphatis zinci, *solutio* muriatis barytæ, &c.

A solution of the *whole substance* in alcohol, or rectified spirit of wine, is sometimes called *tinctura*, and in some similar cases *alcohol*. *Alcohol ammoniatum* and *tinctura camphoræ*. If *tincture* of camphor be proper, the oleum camphoratum should be oleum *camphoræ*, as the last is a solution of the *intire substance*, called camphor, in olive-oil.

I do not perceive any peculiar force in employing the same word as an adjective in one situation and as a substantive in another. Why should there be in this volume tinct. *saponis* and empl. *saponaceum*; tinct. *opii* and pil. *opiatæ* acet. or syr. *scillæ* and pilul. *scilliticæ*?

Trochisci *gummosi* derive their title evidently from the gum arabic, which forms the most material part of their composition. Should not the *emulsio* and the *mucilago*, which also are made of the same gum, have the epithet *gummosa* bestowed upon them, in place of *arabica* to the first, and *mimosæ niloticæ* to the mucilage?

As there is no such thing as gum arabic in the emplastrum *gummosum*, there is room for some other variation to this title; and, were I disposed to invent a substitute, I should, probably, make choice of emplastrum *galbani*. The predominant smell alone would naturally give it the prepre-eminence; and those who think it proper may, if they please, add *compositum.*

The very same denomination is adopted by all the three colleges to signify a medicine, in which the ingredients employed by each are shamefully dissimilar.

It is, or ought to be, thus prepared, wherever the jurisdiction of the London College is in force, viz. of equal parts of *scammony* and *hard extract of jalap,* with one ninth of the whole of *ginger.*

Within the influence of the Edinburgh College, the powder must be composed of equal parts of *scammony* and *cream of tartar.*

And in every corner of Ireland this composition is to be prepared with equal parts of *scammony* and *sulphate of potash,* with one ninth of the whole of *ginger.*

Although no two of these powders are alike, neither in taste nor efficacy, yet they all bear the same title, viz. *pulv. scammon. compositus!*

The words *confectio* and *electuarium* have so long been confounded that they must now be considered as perfectly synonymous. *Confectio* opiata of one college ought therefore to be precisely the same, at least as far as the quantity of opium is concerned, with the *electuarium* opiatum of the other. We find, however, this is not the case, for the first contains one grain of opium in *thirty-six,* and the latter has no more opium in *forty-three* grains of this compound. These two compositions differ also very materially, both in taste, smell, and, perhaps, in other qualities. The electuar. opiat. wants precision, as there is a *sufficient quantity* of wine prescribed; this quantity is consequently left to the discretion of the operator.

There is another instance of considerable disparity, such, however, as may injure the character of the apothecary more than the health of the patient. Each of the dispensatories has a prescription for the aromatic *confection* or *electuary,* so distinctly varied, that the patient may frequently be in doubt, whether from merely changing his residence, and the consequent change of *taste, smell,* and *colour* of the medicine, some blunder has not been committed; and that what he formerly took in Dublin was not at all like the same mixture, draught or bolus, which may now be prepared in London or Edinburgh.

The old *crude antimony* and the *golden sulphur* of *antimony* are now separated in name, merely by the word *præcipitatum,* which, when abbreviated, may be easily mistaken for *præparatum* or even *pulverisatum.* As the dose of of the former, according to the most accurate posological tables, may be extended, I believe with impunity, to *two drachms,* while that of the *golden sulphur* should not exceed *five grains,* and then is frequently attended with very sensible if not violent effects, I should therefore apprehend these have not been sufficiently distinguished from each other.

The same word, *præparatum,* in various other instances, has no meaning whatever. Thus, carbonas ferri and carbonas ferri *præcipitatus.* The respectable translator of the Edinburgh Dispensatory seems also to think it redundant in respect to sulphur *præcipitatum,* since, as a medicine, it is no better than the sulphur in flowers.

There seems no more occasion to put *sublimatum* and *lotum* to the title of sulphur, than to add *solutum, exsiccatum,* and such appendages to *potassa.* Many instances occur of these superfluities in modern pharmaceutic language.

In your Journal for July, I pointed out a case of such dangerous disparity, that I am prompted to take every opportunity to make it known. It is an error of such consequence to society, that I hope every effort will be exerted to rectify the mistake, or at least to put every practitioner on his guard. What were formerly, and, I may say, for ages, known by the names of CALOMEL and that of CORROSIVE SUBLIMATE OF MERCURY, are, agreeably to the modern pharmacopœias, confounded under the *same* title, viz. *murias hydrargyri !!!*

Thus, in Dr. Swediaur's Pharmacopœia, we find it is *calomel;* and, in the Edinburgh Pharm. the hydrarg. muriat. is the strong poison or *corrosive sublimate of mercury.*

Under the head *pilulæ,* many improprieties occur, which should be corrected and revised. The College of Edinburgh avowedly mean to give such names to the formulæ as may rather display the *contents* than the *virtues* of any composition. One would not imagine that in *pil. aloës cum colocynth.* the *most active* ingredient of the whole pill is omitted in the title. If there be eight parts of aloes there are also eight parts of *scammony* in the compound; and as this is by far the most potent, it ought to have occupied the *first* place.

The *aloetic pill* of the three dispensatories is at complete variance with each other. The London College, indeed, adds the word *compositæ,* which, in many other instances, has no precise meaning whatever, especially in the Edinburgh Pharmacopœia.

The

The *opium pill,* besides being discordant in point of power, as it now stands in our Dispensatories, is also not so manageable as it might have been. With extract of liquorice, it frequently becomes tough and indurated, and, consequently, not so miscible with other ingredients with which it is frequently joined.

*Alcohol ammoniatum* is a *binary* compound, consisting of ammonia and alcohol; *oleum ammoniatum* should, if the nomenclature were consistent, be also of the same class. The last is evidently a *ternary* composition, for, besides oil and ammonia, it contains a notable quantity of water.

The copulative *et* and preposition *cum,* as I formerly noticed, are not employed with sufficient precision. *Cum calce* in one case is with lime in *substance,* and in another it is with lime water or in *solution,* viz. potassa *cum* calce, in one instance, and oleum lini *cum* calce, in the other.

The word *oxydum* is often redundant in the nomenclature; and in numberless instances, it may be dropped with propriety. I see no absolute necessity to employ it in the new drawling title for emplastrum lithargyri, viz. *emplastrum oxidi plumbi semivitrei.* Is it possible to vitrify this metal *before* it has become oxidated? It may also be asked how the exact aliquot part of the vitrification is to be ascertained, to warrant such a title? Besides, after the lead has combined with the oil, it is assuredly no longer *litharge;* the water with which this plaster is made contains lead in *solution,* and litharge is *not* soluble in water. If the adjective *album* were added to *emplast. plumbi,* the name perhaps would be more acceptable to those who will not permit the word *litharge* to remain.

The *oxydum plumbi album,* or cerussa of the London College, is a *carbonate.* The oxide of lead attracts carbonic acid almost with as much avidity as potassa does. If this supposed oxyde be put into dilute nitric acid, the doubt will soon be removed by the assistance of a pair of scales, and, perhaps, a little lime water. I am thus explicit, as I have been publicly asked the question.

I am sorry to observe the word *linimentum* is now expunged. The beauty and richness of language often consist in a limited variety of synonymous words. It certainly appears awkward to order a limb to be anointed with a *tincture;* or to bathe the part affected with *tincture of soap* instead of *soap-liniment* or opodeldock.

From the nature of my occupation, I am compelled to a miscellaneous mode of composing these remarks; for, in a former paragraph I ought to have noticed, that although the word *oxydum* be frequently superfluous, it is also as often often left out. This is a clear indication of the capricious state of chemical nomenclature. Among many other cases, we may bring forward several among the class of salts.

Thus, *pot-ash* and *sulphuric acid* form a true *binary* salt, called *sulphat* of *pot-ash;* but *iron* and the same acid can never produce a *sulphat* of *iron.* Nevertheless, to avoid the tedious verbosity of the present system, *I* am satisfied with *sulphas ferri* as a name. It must however be granted, that as the metal must be combined with a portion of oxygen before it be *sulphatised,* the College of Edinburgh and Dr. Swediaur would have been more consistent with the original plan, had they adopted sulphas *oxydi* ferri, murias *oxydi* hydrargyri, sulphas *oxydi* zinci, &c.

Were such rules to be followed up, we might get rid of the word *sub,* which, I believe, is often misapplied. So that *sub*-acetis cupri, according to the new system, might be *oxydum* et acetis cupri, or acetis cum *oxydo* cupri, and boras sodæ *cum sodâ,* with every other *sub*-salt in the whole list.

The article of commerce called *verdegris,* often contains some *metallic* copper, but it chiefly consists of an oxide of the metal, together with some acetic and other vegetable acids, husks and stems of grapes and other adventitious matters, for no two samples are alike; to such a complex mixture I should deem *sub-acetite* of *copper* a very improper and unsatisfactory title.

Each of our Colleges has not only a different name for *sulphuret* of *pot-ash* or liver of sulphur, but orders this compound to be prepared with a variety in the proportion of the ingredients. That by the Edinburgh Pharmacopœia is unnecessarily expensive; there is no occasion to take *pure* pot-ash, as when a carbonate is employed the acid is invariably expelled.

*Pilulæ Rufi, pulvis Doveri,* and some few others, in honour of the inventors, should be suffered to remain. Pulvis ipecac. *compositus* gives one the idea that this powder is a *stronger emetic* than ipecacuanha alone: and pulv. ipecac. *et* opii, to those who are about to commence their study, will never indicate a *sudorific.* Indeed, *P. sudorificus* appears to me sufficiently accurate; and *Doveri* would be no disgrace to the title.

There ought to have been a regular process for *soap,* fit for medicinal purposes, in all the dispensatories; and, were I to prefer one to another, I should take *olive* oil to be very superior to that of almonds, than which nothing is more susceptible susceptible of rancidity; and, in respect to the alkali, we should insert pure *soda* in preference to potassa.

A kind of strict analogy ought to pervade every thing in a pharmacopœia, especially in nomenclature; and the acidum *succini* should, therefore, become *succinicum,* to agree with the whole class of acids.

The process for *sulphas sodæ,* of the Pharm. Edinb. would be more productive, and the crystallisation more perfect and successful, were *carbonate* of *soda* substituted for *chalk,* with a view to saturate any super-abundant acid. Indeed, a small excess of the alkali might be allowed.

The reasons given for using an *excess* of sulphuric acid in the process for *acidum nitrosum,* do not appear conclusive; for, by proper address, a *neutral* sulphat of pot-ash may be abstracted from even a glass retort, with as much security as from an iron pot. This acid may be drawn perfectly pure, at least as *nitrous* acid, from iron vessels; and should sulphuric acid arise, it must be on account of its original excess, or the ignorance of the operator.

In the Medical and Physical Journal for April, I gave an opinion on the oxide of antimony called *pulvis algarothi,* that it is "the most *uniform* oxide" for pharmaceutical purposes. This has been denied in a very respectable, although *anonymous,* quarter, in which it is stated to be a *sub-muriate of antimony.* I can only say, in every analysis, by the most accurate of practical chemists, it has been generally relied on as an *oxide* to ascertain the quantity of antimony contained in any mineral or metallic ore; and relying, as I do, on the repeated experiments of Klaproth, (see his Analytical Essays) I cannot forego that opinion.

As to the new fantastical name for *pulv. antimonialis,* and what I said upon the preparation, it must be recollected, the whole paragraph consisted of interrogatories; I have of course given *no* opinion on that head, but rather wait for information from others. If there be any muriatic acid in the above oxide, it must be considered more as *fortuitous,* than essential to its constitution: Thus there is such a thing as pure potash; but no quantity of lime whatever will abstract the *whole* of the carbonic acid from carbonate of potash; nor will any quantity of water succeed in divesting muriate of antimony of the *whole* of its acid.

The powder projected by Mr. Chenevix, as a substitute for James's powder may, as a medicine, be equal, if not superior to the original. However, as the first is *intirely soluble* in various acids, while the latter remains in part *insoluble*

*insoluble* in the same menstrua, the comparison, in regard to *nomenclature,* must cease.

For my own part, I am surprised the Edinburgh college did not adopt Mr. Chenevix's powder, since there is a degree of neatness and precision in the formula, which must always produce at least an *uniform* preparation; and this cannot be obtained in the present pulv. antimonialis, or *oxydum antimonii cum phosphate calcis.*

In a former communication I remarked, that *purified* opium was in respect to *crude* opium, as 12:9. This was not originally asserted by me, therefore does not bind me down as an advocate for *purifying* opium, which has been insinuated. The London college have, however, continued to trust this *purified* opium for many years; and I confess, although perhaps the process might be dispensed with, I have yet heard no complaints. There is a remark in the English translation of the Ph. Londinens. taken from "*Apothecaries' Report,* that [illegible]/12 of dried opium were taken up by *proof spirit*; and what remained, we may presume, was chiefly feculence. If such a solution be evaporated with *proper care,* the above conclusion will, I apprehend, prove to be very accurate.

Notwithstanding such respectable authority, I am inclined to prefer *crude* opium; for, although the purifying of this very important drug does not *spoil* it, as your correspondent has asserted, it certainly is unnecessary. What the London College term *hard* extracts should be discontinued; for I fear they are seldom to be trusted.

From repeated experiments I have found, that *crude* opium may lose by drying as much as *one-fourth* of its weight. When fresh and newly imported, it is often so very soft as to be unfit even to form pills, unless the operator, at his own discretion, add a little of some innocuous powder, such as starch or liquorice root.

As the several gradations from this extreme to perfect siccity must be very numerous, the compilers of the Edinburgh Dispensatory should, in all cases whatever, have especially insisted upon crude opium in *fine powder,* which would have effectually obviated an inconstancy which must otherwise, I fear, often occur; therefore, *crude* opium, in *fine powder,* implies, consequently, that it must previously be *dried*; and this may be done by a heat not exceeding the common temperature of our summer.

It has ever appeared to me, that there is less danger in preparing medicines to their full extent of power, provided they do not exceed the limits or capacity of the prescription, than than to retrench that efficacy, by lessening the quantity or quality of any important ingredient. Besides *opium*, such formidable medicines as *digitalis, mercury, antimonial* preparations and others, should in all cases be administered in the state of *utmost* strength. Change of residence, to my knowledge, has been often productive of much mischief. One case, in particular, occurs to me, which may serve to elucidate this fact. It was of a lady, who, from the imperfection of *digitalis* in powder, had been ordered to augment the dose. When her medicine was expended, a fresh supply was provided, which, being newly gathered and pulverized, produced effects so much more powerful as to have nearly proved mortal.

One very cogent reason for retaining a great number of old and arbitrary names, such as *litharge, alum,* &c. is the intolerably turgid verbosity of many titles lately adopted. Almost every formula is announced by a bombastic periphrasis. Can any thing be more grating to common sense, than an *oxidum antimonii, cum sulphure per nitratem potassæ,* for crocus antimonii; *oxidum hydrargyri rubrum per acidum nitricum,* for hydrarg. nitrat. ruber; *oleum volatile corticis flavi citrus aurantii,* for oleum corticis aurantii; or, *oxidum stibii hydro-sulphuretum rubro-fuscum,* which is the last and improved edition for *Kermes mineralis.*

To complete the measure of modern absurdity, lest this periphrastic circumlocution should not be understood, Dr. Swediaur has prefixed a species of glossary to each formula, and this immediately succeeds the title. As these lists are in general most grossly imperfect, and more likely to mislead than inform the reader, I sincerely hope they will never be permitted to appear in any British pharmacopœia. In your Journal for July, I selected only a few instances, out of a very extensive number, to shew how far that gentleman's work ought to be trusted for accuracy on this score, as well as on others. I know my observations are generally at variance with at least one critique I have noticed; but that, in my mind, is of little consequence, for society will be more benefited by exposure of *faults,* than dwelling on the *perfections* in books of pharmacy, chemistry, and of every branch of medicine.

On the subject of nomenclature much may be said. The most prominent feature of the chemical system seems to be exceptionable, viz. oxygen; and this, the very keystone of the whole arch, seems defective. When a name is contrived for any newly discovered substance, it should,

 if if possible, be divested of all ambiguity, and applied where, in all cases, it must fit without exception.

It may be insisted on, that several anomalies militate against this name; many things have the property of an acid which do *not* contain oxygen; others, in which it abounds, are *not* acids. Can any thing more effectually neutralize an alkali than common sand or *silex*, in that composition called *glass*, which cannot be decomposed by any acid whatever, if we except the *fluoric*? It is here to be remarked, that, as far as we know, fluoric *acid* contains *no* oxygen; and that this word, from its very beginning, is gratuitously received.

Had the framers of the new system called this very important simple substance *hydrogen*, there could not have been a single exception. As water forms such an immense portion of this habitable globe, and as it contains in every hundred parts, *seventy-five* of this same substance, I am surprised there should have been the least hesitation in deciding the question.

On the present occasion, however, this subject must go no farther; what I have offered may possibly induce others to attend to it, and, by general consent, bring about that speedy and effectual reform, which is so essential to the interest of society in general.

I am, &c.

*Logn Acre, Dec.* 14, 1804. JOS. HUME.

---

*On receiving the following Case of Gout, treated on the cold Water Plan, we doubted whether the Author had correctly applied Dr. Kinglake's Mode of Treatment, and therefore a Copy was sent to Dr. K. for his Opinion, which is subjoined to the Case.*

---

